# Two’s company, three’s a crowd: Social situations alter group dynamics in the maritime earwig (*Anisolabis maritima)*

**DOI:** 10.1371/journal.pone.0343830

**Published:** 2026-03-10

**Authors:** Michael Nolan-Tamariz, Vikram K. Iyengar

**Affiliations:** 1 Warnell School of Forestry and Natural Resources, University of Georgia, Athens, Georgia, United States of America; 2 Department of Biology, Villanova University, Villanova, Pennsylvania, United States of America; Sao Paulo State University Julio de Mesquita Filho: Universidade Estadual Paulista Julio de Mesquita Filho, BRAZIL

## Abstract

Sexual selection can lead to dimorphic traits that can affect an individual’s behavior including how individuals respond to different social situations. We examined how sex and body size influence aggression and courtship in different-sized groups of the maritime earwig, *Anisolabis maritima* (Order Dermaptera), an insect in which males and females differ morphologically and behaviorally. In this study, we videotaped mixed-sex pairs and trios of earwigs for two hours to determine the effects of sex and size on their interactions. We found that, in pairs, females were more aggressive than males and both sexes show size-based aggression. However, sex was a stronger determinant of aggression in male-biased trios whereas sex and size were more important in female-biased trios. Male size did not affect courtship in pairs, as large and small males were equally likely to engage in copulatory activity. The presence of intrasexual selection altered the results in the two types of trios. In male-biased trios, the larger male had more copulatory opportunities as his size relative to his rival increased, indicating that intrasexual domination by larger males can overcome female preferences for smaller males observed in previous studies. Interestingly, in female-biased trios, there were no such patterns since copulatory opportunities were relatively rare, the likely result of elevated aggression with two interacting females. Our results indicate that sex and size affect both aggression and sexual behavior differently based on group size and composition, which highlights the need to examine behaviors in a variety of social contexts.

## Introduction

Sexual selection involves differential reproductive success regarding competition over access to mates [1,2]. This competition may involve intersexual interactions (such as male courtship and female mate choice), intrasexual interactions (such as male-male combat), or a combination of both [3,4]. Strong selective pressures from each type of interaction can lead to the development of sex-specific traits that lead to a sexual dimorphism where male ornamentation and weaponry are reproductively advantageous [5]. For example, across a wide variety of taxa, females mate preferentially with males possessing elaborate ornaments that are indicative of quality [6–8]. Weaponry has also been observed in many species, where such traits provide a competitive advantage in direct competition with rivals for access to potential mates or essential resources [9–11]. Although males typically develop these combative traits, in some species weaponry has also been observed in females (either sex role reversal or along with males) [3,12], which leaves open the possibilities for intersexual choice and intrasexual competition in both sexes [2].

Because sexual selection often involves varying degrees of both inter- and intrasexual selection, it is often difficult to separate and distinguish these factors and gauge their relative contributions to a particular mating system. Typically, in most species, larger males obtain more matings than smaller males, as female choice-based male quality reinforces male competition outcomes [13–16]; however, this is not always the case and there may be antagonistic selection where particular traits have multiple selective pressures that interact [17–20]. In fact, females may actively select against traits that aid males in intrasexual competition; for example, female carrion flies prefer males with elongated heads that are at a disadvantage in intrasexual competition [21], and female marbled gobies prefer smaller males in the absence of male competition [22]. Additionally, mate choice can also be based on morphology where the optimal male mate may depend on female state and size, which may lead to assortative mating where individuals of both sexes choose larger mates due to reproductive benefits, leaving smaller, less desirable individuals, left to pair up [23–25]. Given the differences in how individuals interact between and among the sexes, it is important to study how sexual selection can vary in aggregations based on group size and composition [26].

Here, we investigated the role of group size and composition (i.e., the size and sex of participants) on aggression and courtship in the maritime earwig, *Anisolabis maritima* (Order Demaptera, Family Anisolabididae). This insect is found worldwide on temperate and tropical beaches at or above the high tide line, where they aggregate under driftwood or seaweed and emerge only at night to forage on small arthropods [27,28]. Although both sexes have weaponry, *A. maritima* is sexually dimorphic regarding its most distinctive feature in that females have straight posterior forceps (pincers) whereas males have asymmetrical, curved forceps that are used in prey capture and intraspecific competition [29,30]. Males and females also differ fundamentally in their aggression during agonistic encounters. Females lay eggs and violently guard their nest, often killing conspecifics by using their straight forceps to strike and cut opponents into pieces whereas males typically resolve their disputes non-lethally by squeezing each other, perhaps as a means to assess strength and fighting ability [30–32]. During the breeding period, it is common to encounter multiple nesting females beneath a refuge with only one or two males present nearby (personal obs.), a pattern consistent with a polygynous mating system in which certain males associate with, or potentially monopolize, groups of females. As in many insects, female maritime earwigs are typically larger than males [27,33], but male size may follow a bimodal distribution, with approximately 20% of males being significantly larger than the average female, similar to other earwig species such as the European earwig, where a male’s forceps morphology and size is largely dependent on nutrient availability during the nymph stage of development [34]. In this earwig species, the frequency of unusually large males has been shown to be related to a variety of environmental factors, including population density [35].

Previous work in *A. maritima* shows that both intersexual and intrasexual selection can affect interactions and behavioral outcomes. Sex-based aggression is important in determining the spacing of individuals, as increased female presence and their higher levels of aggression lead to less cohabitation and greater spacing among individuals [36,37]. Larger individuals of both sexes are more aggressive and have a competitive advantage in intrasexual battles, which often leads to higher fitness through access to food and mates [30,32,38]. However, although male size plays a large role in intrasexual competition for females, females control copulation since they also have weaponry and can terminate the mating as any time [39]. Indeed, males are vulnerable during courtship, which involves their genitalia emerging between their forceps as they move backwards towards the forceps of the female, whose genitalia are also located in the same region as their weapons [40,41]. Males risk damaging their reproductive organs every time they court, and, within many populations, over half of the male genitalia are damaged [42]. Since females have weapons and must orient themselves in a specific manner allow for mating, any copulatory activity (i.e., courtship) is the product of both male interest and female receptivity and should be considered female choice. Finally, there is also more direct evidence that female choice plays an important role in this mating system, as females have been shown to mate preferentially with smaller males when given the opportunity [37]. Although small females can be coerced into cohabitating with much larger males, females will typically choose to cohabitate with smaller males, perhaps due to reduced risk of injury during mating [43,44]. Thus, both sex and the size of individuals affect the nature and outcome of interactions in *A. maritima*.

In this study, we examined the role of sex and size in the mating system of *A. maritima* by creating intersexual pairs and trios where we continuously recorded interactions representing aggression (strikes), copulatory activity (forceps-to-forceps contact), and neutral activities (antennation and incidental contact). We expected that larger individuals would be more aggressive in both sexes due to their intrasexual competitive advantages and that females would show higher levels of aggression than males. We also hypothesized that there would be different levels of aggression and copulatory activity in pairs vs. trios. Specifically, we predicted that adding another individual would increase aggression and decrease copulatory activity to the presence of another individual, either by increasing the overall aggression due to adding another weapon-bearing individual or by virtue of introducing intrasexual competition. We also predicted more frequent copulatory activity in pairs, where females would be more likely to be receptive to smaller males in the absence of intrasexual aggression and competition. We expected that not only would there be less copulatory activity in trios, but also that there would be differences between the two types of trios in that adding a (more violent) female would lead to more aggression and less copulatory activity relative to adding a (less violent) male.

## Materials and methods

### Study organism and morphological measurements

We collected all individuals under driftwood on beaches on San Juan Island, WA with permission of the University of Washington’s Friday Harbor Laboratories in the summer of 2016. Each individual was housed alone in a 0.5L glass jar with moistened sand and wet cat food to reduce the likelihood that interactions were influenced by recent mating, desiccation, or hunger. Trials were conducted 24–48 hours after capture to ensure no mating took place the day prior and to allow for acclimation to lab conditions. We used each individual in only one trial, after which it was frozen for subsequent morphological measurements [36–38].

We photographed each individual with a SONY CCR-DC374 digital camera attached to a Nikon SMZ800 and used ImageJ 1.48v to measure the pronotum width at its widest point as an index of size [30]. We also calculated a focal individual’s size relative to its opponent, defined as $RS=\frac{f-o}{f}\;\times \;\text{1}\text{0}\text{0}$, where *f* is the focal individual’s pronotum width, and *o* is the opponent’s pronotum width. Both size and relative size were treated as continuous variables.

### Experimental trials

We conducted experiments across three different trial types: (a) Pairs, (b) 2M1F Trios, and (c) 2F1M Trios. For pairs, we placed one randomly selected male and one randomly selected female into plastic containers (8.5 cm x 13 cm x 7.5 cm) with 0.5 cm of wet sand collected from False Bay, WA. Marking was not necessary as males and females were easily distinguished visually by the shape of their forceps. For trios, we placed two randomly-selected, same-sex individuals with a randomly selected opposite-sex individual (hereafter referred to as the lone individual). Half of such trials involved two males and one female (2M1F Trios), and half involved two females and one male (2F1M Trios). These trials took place in larger plastic containers (12 cm x 17.5 cm x 10 cm) that contained wet sand and two red-tinted plastic squares (5 cm x 5 cm) placed on opposite ends of the arena to serve as shelters [36]. We marked the same-sex individuals with one or two dots of white out placed on the pronotum to distinguish between them without altering behavior [36].

We recorded trials using a LogitechTM C920 webcam placed approximately 45 cm above arenas and Apple PhotoBooth (version 8.0). Following each trial, we cleaned and refilled arenas with new sand to remove any potential trace of pheromones. Trials lasted two hours (2100 h to 2300 h), as most hierarchies are determined within approximately 25 minutes [30]; extending trials beyond this window ensured sufficient opportunity for interaction and contest resolution. Trials were conducted under red light to allow for adequate viewing while maintaining perceived darkness for the earwigs. We classified all interactions as one of the following: incidental contact, offensive strike, defensive strike, antennation, and forceps-to-forceps contact (S1 Table).

### Statistical analyses

**Measuring aggression.** To quantify aggression, we used zero-inflated generalized linear mixed models (GLMMs) with a beta distribution and logit link in the glmmTMB package in R (version 4.4.2) [45]. These models include both a zero-inflation portion, to account for excess zeros, and a conditional portion to evaluate non-zero values. The response variable was the proportion of aggressive acts, defined as the number of total strikes divided by the total number of interactions. We included Trial (in pairs and trios) and individual ID (in trios only) as random effects to account for repeated measures.

We built two types of candidate models. Each included sex and either size or relative size as fixed effects. Due to high collinearity (r > 0.7), size and relative size were never included in the same model [46]. For each type of candidate model, we tested two variants; one included only main effects while the other also included their interaction. The same fixed effects were used in the zero-inflation and conditional portions. We compared both variants of each candidate model using Akaike’s Information Criterion (AIC), and we report the model with the lowest AIC for each trial type.

**Mate preference.** We classified individuals’ size as either “small” or “large” based on post-trial morphological measurements. In Pairs and lone-sex individuals in Trios, individuals were categorized using the median of the sex-specific distribution of pronotum widths, with each trial type treated as a separate population. This approach allowed us to test if copulation rates differed among individuals relative to others within the same population.

For same sex-individuals in Trios, we classified all individuals based on their pronotum width relative to the same-sex opponent. The individual with the wider pronotum as “larger” and the individual with the narrower pronotum width as “smaller”. Prior work and our preliminary analyses indicate that body size is a key driver of copulatory activity [36–38]. Accordingly, because our objective was to test if copulatory rates differed between differently sized same-sex opponents, we excluded trials where same-sex individuals differed in pronotum width by ≤ 5%, thereby restricting analyses to trials with clear same-sex size differences.

Copulatory activity was assessed as a binary outcome based on the occurrence of forceps-to-forceps contact during each trial [40]. To test for preferential copulatory activity, we used availability weighted chi-square goodness-of-fit tests to compare the frequency with which individuals in each size class engaged in copulatory activity relative to their representation in the population. In Trios with differently sized same-sex opponents, each trial contributed one larger and one smaller individual of the same sex, and either one large or one small individual of the lone sex to the expected frequencies. For example, across 30 size-asymmetric 2M1F Trio trials, expected availability includes 30 larger and 30 smaller females, and approximately 15 large and 15 small males.

To account for small, uneven sample sizes, we used Monte Carlo simulation (10,000 replicates) to assess statistical significance. Separate tests were conducted for each sex in each trial type. All chi-square tests were conducted in base R (version 4.4.2) [45].

**Predicting copulatory behavior.** To identify the predictor variables most strongly associated with the occurrence of copulatory behavior, we conducted stepwise selection separately on two sets of predictors: size and behavioral variables. Copulatory behavior was modeled as a binary response (presence or absence of forceps-to-forceps contact) using generalized linear models (GLMs) with a binomial family and a logit link. For each trial type (Pairs, 2M1F Trios, and 2F1M Trios), we began with a null model including only an intercept. Full models varied by predictor set (size or behavior) and trial type and did not include interaction terms. All trials were retained for these analyses, including those previously excluded from mating preference analyses.

In Pairs, the full size model included size and relative size of both individuals. In 2M1F Trios and 2F1M Trios, the full size model similarly included size and relative size (relative to both opponents) of all three individuals. In Trios we refer to individuals as “larger”, “smaller”, and “lone” solely for bookkeeping purposes to track individual identity and directionality in dyadic interactions (e.g., direction of strikes). These labels were assigned post-trial based on morphological measurements and are analogous to designations such as Male 1/Male 2 or Female A/Female B. They were not intended to imply categorical size classes across trials as both size and relative size were treated as continuous variables for all individuals in all models.

For behavioral predictors in Pairs, the full model included antennation, incidental contact, and offensive, defensive, and total strikes (sum of offensive and defensive strikes) for both sexes. In 2M1F and 2F1M Trios, the full behavioral model included antennation, incidental contact, and offensive, defensive, and total strikes across all dyadic combinations (larger-smaller, larger-lone, and smaller-lone) with directionality explicitly accounted for (larger-to-smaller, larger-to-lone, smaller-to-larger, smaller-to-lone, lone-to-larger, and lone-to-smaller).

Before constructing candidate models, we assessed multicollinearity among the predictors retained via stepwise selection and excluded variables with pairwise correlations exceeding r > 0.7 [46]. This process resulted in two notable outcomes. First, directional relative size measures within dyads (e.g., larger male-to-female and female-to-larger male) were highly correlated, and we therefore retained only a single relative size term per dyad. Second, offensive, defensive, and total strikes were also highly correlated, and we consequently retained total strikes as a composite measure of aggressive behavior.

We then constructed sets of candidate GLMs that combined size and behavioral predictors. For each trial type, candidate models were compared using AIC and we report the effect estimates from the model with the lowest AIC.

## Results

### Measuring aggression

For each trial type, we evaluated two candidate models with two variants, resulting in four total models. Across all trial types, models that included relative size had lower AIC values than those that included size. ΔAIC ranged from 2.604 to 12.488 (Table 1), indicating relative size was a better predictor of aggression in our data. Within the sets of models that included relative size, those without an interaction between relative size and sex had lower AIC values than those that did (Table 1). However, for pairs and 2M1F trios, the ΔAIC between interaction and non-interaction models was < 1, suggesting these models were comparable. In contrast, the ΔAIC for the 2F1M Trios was 3.926, providing somewhat stronger support for the non-interaction model. Importantly, while both variants show similar patterns (with one noted exception) the interaction between relative size and sex was not statistically significant in any model. For these reasons, we report below the results of the relative size and sex models without the interaction term; the corresponding results for models including the interaction are provided as supporting information under S2 Table.

**Table 1:** Comparisons of zero-inflated beta mixed-effect regression models examining the effects of size, relative size, sex, and their interactions on aggression in (A) Pairs, (B) 2M1F Trios, and (C) 2F1M Trios of *A. maritima* on San Juan Island, WA in 2016. Model formula reflects both the zero-inflation and conditional portions of the model.

A) Pairs			
Model Formula	df	AIC	ΔAIC
Relative Size + Sex	8	57.517	0
Relative Size + Sex + Relative Size * Sex	10	58.032	0.515
Size + Sex	8	68.285	10.768
Size + Sex + Size * Sex	10	70.005	12.488
B) 2M1F Trios			
**Model Formula**	**df**	**AIC**	**ΔAIC**
Relative Size + Sex	9	36.746	0
Relative Size + Sex + Relative Size * Sex	11	36.747	0.001
Size + Sex	9	39.350	2.604
Size + Sex + Size * Sex	11	40.066	3.320
C) 2F1M Trios			
**Model Formula**	**df**	**AIC**	**ΔAIC**
Relative Size + Sex	9	76.695	0
Relative Size + Sex + Relative Size * Sex	11	80.621	3.926
Size + Sex	9	87.468	10.773
Size + Sex + Size * Sex	11	88.168	11.473

In pairs (N = 59), males were more likely to produce values of zero aggression, but there was no significant difference in the levels of aggression between males and females in the conditional portion of the model (Table 2A). Relative size did not have a significant effect in the zero-inflation portion, although relatively larger individuals showed higher levels of aggression in the conditional portion of the model (Table 2A).

**Table 2:** Results from zero-inflated beta mixed-effect regression models examining the effects of relative size, and sex on aggression in (A) Pairs, (B) 2M1F Trios, and (C) 2F1M Trios in *A. maritima* on San Juan Island, WA in 2016. Proportion of aggressive acts, defined as the number of strikes divided by the number of interactions, was the response variable.

A) Pairs (AIC = 57.517)						
Zero – Inflation Portion	**Coefficient**	**Odds Ratio**	**Estimate**	**SE (Estimate)**	**Z Value**	**P Value**
	Intercept	0.088	−2.434	0.524	−4.646	< 0.001
	Relative Size	0.967	−0.033	0.017	−1.955	0.051
	Sex (Male)	7.115	1.962	0.610	3.214	0.001
Conditional Portion	**Coefficient**	**Odds Ratio**	**Estimate**	**SE (Estimate)**	**Z Value**	**P Value**
	Intercept	0.403	−0.910	0.149	−6.112	< 0.001
	Relative Size	1.018	0.018	0.006	3.043	0.002
	Sex (Male)	0.747	−0.292	0.214	−1.365	0.172
B) 2M1F Trios (AIC = 36.746)						
Zero – Inflation Portion	**Coefficient**	**Odds Ratio**	**Estimate**	**SE (Estimate)**	**Z Value**	**P Value**
	Intercept	0.400	−0.916	0.288	−3.177	0.001
	Relative Size	0.975	−0.025	0.009	−2.809	0.005
	Sex (Male)	2.424	0.885	0.345	2.567	0.010
Conditional Portion	**Coefficient**	**Odds Ratio**	**Estimate**	**SE (Estimate)**	**Z Value**	**P Value**
	Intercept	0.195	−1.633	0.143	−11.416	< 0.001
	Relative Size	1.007	0.007	0.005	1.481	0.139
	Sex (Male)	0.659	−0.418	0.149	−2.807	0.005
C) 2F1M Trios (AIC = 76.695)						
Zero – Inflation Portion	**Coefficient**	**Odds Ratio**	**Estimate**	**SE (Estimate)**	**Z Value**	**P Value**
	Intercept	0.437	−0.828	0.208	−3.985	< 0.001
	Relative Size	0.948	−0.053	0.011	−4.639	< 0.001
	Sex (Male)	2.697	0.992	0.340	2.917	0.004
Conditional Portion	**Coefficient**	**Odds Ratio**	**Estimate**	**SE (Estimate)**	**Z Value**	**P Value**
	Intercept	0.250	−1.388	0.159	−8.712	< 0.001
	Relative Size	1.014	0.014	0.006	2.367	0.018
	Sex (Male)	0.574	−0.556	0.238	−2.334	0.020

In 2M1F Trios (N = 31), males were more likely to produce values of zero aggression and showed lower levels of aggression than females in the conditional portion of the model. (Table 2B). Relatively larger individuals were less likely to produce values of zero aggression, although we note a discrepancy with the interaction variant where relative size did not have a significant effect in the zero inflation-portion (S2 Table). Relative size did not have a significant effect on aggression levels in the conditional portion of the model (Table 2B).

In 2F1M Trios (N = 32), males were more likely to show zero aggression as well as lower levels of aggression in the conditional portion of the model (Table 2C). Relatively larger individuals were less likely to show zero aggression and showed higher levels of aggression in the conditional portion of the model (Table 2C).

### Mate preference

In Pairs, copulatory activity occurred in 32 of 59 trials (54.2%). Across all trials, large and small males and females were represented at similar frequencies across trials (Table 3), and no size class of either sex engaged in copulatory activity at a rate disproportionate to its representation (males: χ² = 0.599, p = 0.488; females: χ² = 0.931, p = 0.383).

**Table 3:** Descriptive summaries of copulatory activity by size and sex classes in Pairs, 2M1F Trios, and 2F1M Trios in *A. maritima* on San Juan Island, WA in 2016. The data (x/ N) represent the number of trials in which an individual of a size class engaged in copulatory activity (x) relative to the number of trials in which that size class was present (N)*. In 2M1F Trios and 2F1M Trios, there was one instance where both same-sex individuals engaged in copulatory activity within a single trial. In Pairs, females accepted courtship from both large and small size classes at equal rates. However, in 2M1F Trios, females more frequently accepted copulatory activity from the larger of the two males. Chi-square tests in the text reflect individual-level weighted availability.

	Pairs		2M1F Trios		2F1M Trios	
	Male	Female	Male	Female	Male	Female
Large(r)	13/ 28	17/ 29	12/ 30	7/ 15	4/ 12	4/ 26
Small(er)	19/ 31	15/ 30	2/ 30	6/ 15	2/ 14	3/ 26

In 2M1F Trios, size-matched males appeared in only 1 of 31 trials and were excluded from further comparison. Among the remaining 30 trials, copulatory activity occurred in 13 trials (43.3%), with one instance in which both the larger and smaller males engaged in copulatory activity. Large and small lone females were represented at comparable frequencies (Table 3) and neither size class engaged in copulatory activity at a rate disproportionate to its representation (χ² = 0.077, p > 0.999). In contrast, larger males engaged in copulatory activity at a rate greater than expected based on their representation in the sample (χ² = 7.143, p = 0.013).

In 2F1M Trios, size-matched females appeared in 6 of 32 trials and were excluded from further comparison. Among the remaining 26 trails, copulatory activity occurred in 6 trials (23.1%), with one instance in which both the larger and smaller female engaged in copulatory activity. Large and small lone males were represented at comparable frequencies (Table 3) and neither male size class engaged in copulatory activity at a rate disproportionate to its representation (χ² = 1.012, p = 0.431). Similarly, neither size class of females engaged in copulatory activity at a rate disproportionate to its representation in the sample (χ² = 0.143, p > 0.999).

### Predicting copulatory behavior

**Fig 1:**
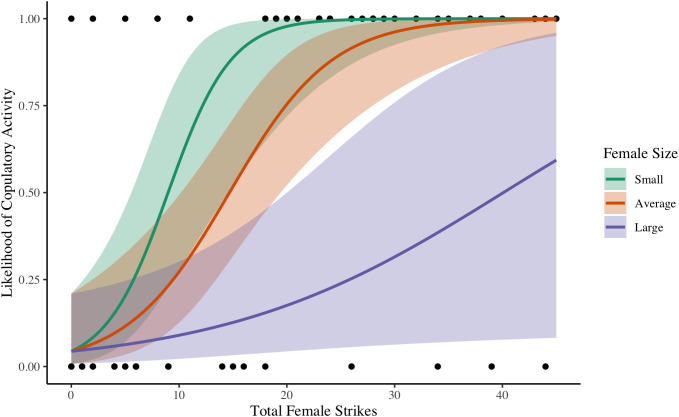
Likelihood of copulatory activity as a function of female strikes and size in Pairs of *A. maritima*, San Juan Island, WA, 2016. This figure visualizes the significant interaction reported in Table 5A, where each point represents the outcome of a single Pairs trial. Predicted probabilities (solid lines) are shown with 95% confidence intervals (shaded areas). “Average” (orange) corresponds to mean pronotum width while “Small” (green) and “Large” (purple) correspond to a pronotum width 2 standard deviations above and below the mean respectively. As female absolute size increases, the effect of strikes on the likelihood of copulatory activity decreases.

In Pairs (N = 59), the predictors identified via stepwise selection were male size, female size, female relative size, antennation, female total strikes, and male total strikes. We constructed 38 candidate models. Among the five most supported candidates, there was a ΔAIC of less than 1 (Table 4A). All five models included antennation, female total strikes, and the interaction of female total strikes and female size. Four of the five models also include male total strikes. The lowest AIC model included significant effects of antennation, female total strikes, and a significant interaction between female total strikes and female size (Table 5A; Fig 1). We report the results of the lowest AIC model below and results of the remaining four most supported models as supporting information (S3 Table).

**Table 4:** Comparisons of generalized linear models of copulatory activity in (A) Pairs, (B) 2M1F Trios, and (C) 2F1M Trios of *A. maritima* on San Juan Island WA in 2016. (F = Female, M = Male, LF = Larger Female, SF = Smaller Female, LM = Larger Male, SM = Smaller Male).

A) Pairs			
Model Formula	df	AIC	ΔAIC
Antennation + Total Strikes (F) + Total Strikes (M) + Total Strikes (F) * Size (F)	5	52.770	0
Antennation + Total Strikes (F) + Total Strikes (F) * Size (F)	4	53.061	0.291
Antennation + Total Strikes (F) + Total Strikes (M) + Size (M) + Relative Size (F) + Total Strikes (F) * Size (F)	7	53.238	0.468
Antennation + Total Strikes (F) + Total Strikes (M) + Size (F) + Relative Size (F) + Total Strikes (F) * Size (F)	7	53.368	0.598
Antennation + Total Strikes (F) + Total Strikes (M) + Size (M) + Size (F) + Total Strikes (F) * Size (F)	7	53.704	0.934
B) 2M1F Trios			
**Model Formula**	**df**	**AIC**	**ΔAIC**
Antennation (LM + F) + Total Strikes (F to SM) + Relative Size (LM to SM) + Total Strikes (F to SM) * Relative Size (LM to SM)	5	29.700	0
Total Strikes (F to SM) + Relative Size (LM to SM) + Total Strikes (F to SM) * Relative Size (LM to SM)	4	33.786	4.086
Antennation (LM + F) + Total Strikes (F to SM) + Relative Size (LM to SM) + Antennation (LM + F) * Total Strikes (F to SM)	5	36.287	6.587
Total Strikes (F to SM) + Relative Size (LM to SM)	3	36.783	7.083
Antennation (LM + F) + Total Strikes (F to SM) + Relative Size (LM to SM) + Antennation (LM + F) * Relative Size (LM to SM)	5	37.393	7.693
C) 2F1M Trios			
**Model Formula**	**df**	**AIC**	**ΔAIC**
Size (LF) + Size (SF) + Relative Size (M to LF) + Relative Size (M to SF) + Total Strikes (LF to SF) + Total Strikes (M to LF) + Relative Size (M to LF) * Total Strikes (M to LF)	8	27.877	0
Size (LF) + Size (SF) + Relative Size (M to LF) + Relative Size (M to SF) + Total Strikes (LF to SF) + Total Strikes (M to LF)	7	28.089	0.212
Size (LF) + Size (SF) + Relative Size (M to LF) + Relative Size (M to SF) + Total Strikes (LF to SF)	6	28.324	0.447
Size (LF) + Size (SF) + Relative Size (M to LF) + Relative Size (M to SF) + Total Strikes (LF to SF) + Total Strikes (M to LF) + Relative Size (M to LF) * Total Strikes (LF to SF)	8	29.148	1.271
Size (LF) + Size (SF) + Relative Size (M to LF) + Relative Size (M to SF) + Total Strikes (LF to SF) + Total Strikes (M to LF) + Relative Size (M to LF) * Size (SF)	8	29.277	1.400

**Table 5:** Results of generalized linear models of copulatory activity in Pairs (A), 2M1F Trios (B), and 2F1M Trios (C) of *A. maritima* on San Jauan Island, WA in 2016. Predictor variables were selected using stepwise selection to construct a set of candidate models. Model performance was evaluated using Akaike’s Information Criterion (AIC), and results are reported for the lowest AIC model in each trial type. (F = Female, M = Male, LF = Larger Female, SF = Smaller Female, LM = Larger Male, SM = Smaller Male).

A) Pairs (AIC = 52.770)					
**Coefficient**	**Odds Ratio**	**Estimate**	**SE (Estimate)**	**Z value**	**P value**
Intercept	0.006	−5.122	1.466	−3.494	< 0.001
Antennation	1.210	0.191	0.070	2.717	0.007
Total Strikes (F)	2.442	0.893	0.310	2.877	0.004
Total Strikes (M)	1.045	0.044	0.029	1.553	0.120
Total Strikes (F) * Size (F)	0.805	−0.217	0.085	−2.559	0.011
B) 2M1F Trios (AIC = 29.700)					
**Coefficient**	**Odds Ratio**	**Estimate**	**SE (Estimate)**	**Z value**	**P value**
Intercept	< 0.001	−22.475	10.062	−2.234	0.026
Antennation (F + LM)	1.798	0.586	0.308	1.906	0.057
Total Strikes (F to SM)	32.707	3.488	1.644	2.121	0.034
Relative Size (LM to SM)	2.291	0.829	0.382	2.167	0.030
Total Strikes (F to SM) * Relative Size (LM to SM)	0.872	−0.137	0.067	−2.040	0.041
C) 2F1M Trios (AIC = 27.877)					
**Coefficient**	**Odds Ratio**	**Estimate**	**SE (Estimate)**	**Z value**	**P value**
Intercept	> 100	75.573	46.180	1.636	0.102
Size (LF)	> 100	101.027	89.789	1.125	0.261
Size (SF)	< 0.001	−129.030	105.145	−1.227	0.220
Relative Size (M to LF)	19.127	2.951	2.525	1.169	0.242
Relative Size (M to SF)	0.035	−3.346	2.766	−1.210	0.226
Total Strikes (LF to SF)	0.663	−0.411	0.320	−1.283	0.199
Total Strikes (M to LF)	2.931	1.075	0.808	1.330	0.183
Relative Size (M to LF) * Total Strikes (M to LF)	1.083	0.080	0.081	0.979	0.328

**Fig 2:**
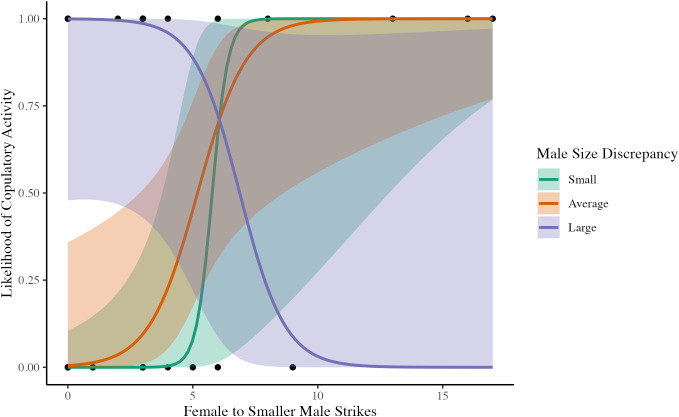
Likelihood of copulatory activity as a function of female-to-smaller male strikes and male size discrepancy (larger male to smaller male relative size) in 2M1F Trios of *A. maritima.* This figure visualizes the significant interaction shown in Table 5B, where each point represents the outcome of a single 2M1F Trio trial. Predicted probabilities (solid lines) are shows with 95% confidence intervals (shaded areas). “Average” (orange) corresponds to mean male size discrepancy while “Small” (green) and “large” (purple) correspond to a size discrepancy 2 standard deviations above and below the mean respectively. As male size discrepancy increases, the effect of female-to-smaller male strikes weakens and eventually reverses.

In 2M1F Trios (N = 31), the predictors identified were larger male to smaller male relative size, antennation between the larger male and female, and female to smaller male total strikes. We constructed 11 candidate models. The lowest AIC model had significant effects of female to smaller male total strikes, larger male to smaller male relative size, and a significant interaction between female to smaller male total strikes and larger male to smaller male relative size (Table 5B; Fig 2).

In 2F1M Trios (N = 32), the predictors identified were male-to-larger female relative size, male-to-smaller female relative size, larger female size, smaller female size, larger-to-smaller female total strikes, male-to-larger female total strikes, smaller female-to-male total strikes, and larger female and smaller female antennation. We constructed 39 candidate models. Among the five most supported candidates, there was a ΔAIC less than 2 (Table 4C). All five models included the effects of larger female size, smaller female size, male to smaller female relative size, and larger female to smaller female total strike (Table 4C). Four of the five models also included male to larger female total strikes and three of the five also included an interaction term (Table 4C). In the lowest AIC model, none of the predictors were statistically significant (Table 5C). We report the full effects of the lowest AIC model below and effects of the remaining four most supported models in (S4 Table).

## Discussion

Overall, we found that social situations, particularly sex ratios in small groups, affect both aggressive behavior and copulatory activity in *A. maritima*. In addition to confirming that females are typically more aggressive than males and that both females and males show size-based aggression, we show how those sex- and size-based differences translate changes in group dynamics based on the presence and ratio of those individuals. Specifically, sex is the primary driver of aggression in the system, but size is the most important factor explaining differences in aggression within each sex. Regarding copulatory activity, while we did not find a size-based difference in male courtship within Pairs, we showed that intrasexual aggression can alter the dynamics and the outcome of mating behavior.

We expected to find differences in aggression based on both sex and size. Females have been shown to have a higher baseline aggression than males [36], perhaps due to fact that they exhibit maternal care and guard their nests against violent conspecific intruders. Previous work has also shown that larger individuals dominate in both intersexual and intrasexual interactions – larger, more aggressive mothers are more successful in defending their eggs against other earwigs [32], and larger males are more likely to win intrasexual battles for food [30] and for mates [38]. This pattern is not surprising because weaponry is proportional to body size [30]; however, our results show how such individual differences affect group interactions and behavioral outcomes. In pairs, when there is only one male and one female, we found that smaller individuals were less aggressive and that males were less aggressive than females. When found in trios, however, the sex composition of the group determined the most important factor affecting aggression. When there were two males and one female (2M1F Trios), sex mattered but size did not affect aggression. This result is likely due to intrasexual interactions between the different-sized males, as the larger males can dominate smaller ones without the need for aggressive strikes – in fact, intrasexual aggression likely de-escalated quickly, as competing males often determine their hierarchy within minutes [30]. Thus, while the level of aggression between the males was likely relatively low, the female’s levels may have been elevated as she tried to fend off courtship advances from one or both males. We posit that there was no effect of size on aggression here because different-sized males often establish a dominance hierarchy quickly [30], which deescalates aggression and the number of strikes exchanged. Interestingly, when there were two males and a female (2F1M Trios), size affected also aggression across our two models. We believe this result stems from the fact that, in these trials, the majority (two of three) of individuals were aggressive females, which would lead to an increased hostility. The difference between the two types of trios highlights how sex differences affect the dynamics of groups based on sex ratio.

Regarding copulatory activity, we anticipated that small or smaller males would be more likely to gain courtship opportunities than large or larger males [37]. Since male copulatory activity requires compliance from aggressive females, this outcome is more likely due to female preferences for relatively smaller males rather than small male competitive advantages. In pairs (with one male and one female), we were unable to confirm this female preference as both size classes of males courted at equal rates. When there was a third individual, however, the copulatory activity among the participants changed based on the sex composition of the trio. In 2M1F Trios, larger males engage in more copulatory activity than smaller males. We attribute this result to intrasexual aggression, with the larger male bullying the smaller male and limiting his opportunities to interact with the lone female. In 2F1M Trios, there were no size-based differences in copulatory activity. Since we found that the copulation rate (20%) was less than half of that observed in the other two types of groups, we hypothesize that the intrasexual aggression between the two females created a violent environment that made it difficult for the lone male to court. The main difference between the trials stem from the effect of intrasexual interactions on the frequency of courtship opportunities and aggressive interactions.

Since females control copulatory activity by having the ability to reject such advances with their weaponry, we were not surprised that female receptivity was unaffected by her own size – it appears that different-sized females have similar criteria for accepting male courtship advances. Neither size class of females was more likely to gain courtship opportunity, which indicates that males will engage in courtship/copulation whenever possible, even with a larger female if she is receptive. Although these findings are contrary to previous findings of preference for a smaller partner in both sexes [37], indiscriminate male courtship with respect to female characteristics is common in polygynous species, where there is an incentive for males to mate with as many females as possible [47]. This general rule does not mean that a male preference for smaller mates does not exist (possibly to minimize risk), rather that the potential reproductive benefits of multiple mates may outweigh the potential cost of interacting with larger, more aggressive females.

Across all the trials, we monitored other types of interactions including neutral interactions (e.g., antennation) and negative interactions (e.g., strikes) to determine their role in copulatory activity. In pairs, we found that females were generally more likely to accept copulatory activity from individuals that they interacted with more, whether it was through non-violent antennation or violent strikes. However, we also found a significant interaction between female strikes and female size that shows that, as female size relative to the male increases, the positive effect of strikes on copulatory activity weakens. In other words, when females are extremely large, either males do not engage with her nearly as often (due to intimidation) or such large females are simply not interested in such puny males (due to their low quality). Overall, regardless of the nature of the interaction, females were more likely to mate with males that were persistent in approaching her (similar to Ross and Iyengar *in prep*). We posit that females use interactions (including strikes) as a way to gauge the quality of a potential mate (motor performance and energetic reserves) [48] or to ensure their potential partner is not too hostile or aggressive (assurance of survival during courtship) [43]. On the other hand, we found no such effect of male interactions on his copulatory activity, which further indicates that male behavior has no effect on courtship success and females dictate copulation.

Compared to the pairs, we found different copulatory activity patterns in trios that we attribute to whether the third individual was male or female. In 2M1F Trios, more female interactions with the smaller male (primarily driven by aggressive strikes) led to a greater chance of copulatory activity, which is the same pattern observed in pairs. However, another interesting pattern emerged in that copulatory activity was more likely to occur as the larger male size relative to the smaller male increased. We posit that this is the result of intrasexual competition; the greater the size discrepancy among the males, the faster the larger male will “win” and the smaller male will back down, which will in turn allow the males to focus on courting the female rather than fighting with each other. Further analyses show that, with the decrease in aggression and strikes concomitant with male size disparities, there is no longer an effect of strikes on copulatory activity for small males. We suspect that such vast size discrepancies mean that these tiny males do not engage with females nearly as often due to being kept away from the female or simply being intimidated by the huge male. It appears that the only situation in which smaller males can court are when the females do not strike the smaller male very much, which implies that females and smaller males are engaging quickly to avoid aggression by the larger male. In 2F1M Trios, on the other hand, we observed no effects of size or aggression on copulatory activity. These results are consistent with our earlier speculation in which we attribute the low frequency of copulatory activity to the presence of multiple females, who are innately more aggressive and whose intrasexual strikes may preclude receptivity to males. Together, the results from the two types of trios further highlight the importance of intrasexual aggression in determining how much energy is available for copulatory activity.

In conclusion, our results demonstrate that the social context affects both aggression and copulatory activity in the maritime earwig. We found that individual differences based on sex and size manifest themselves in changing group dynamics based on the composition of individuals in small groups – that is, in groups of three, intrasexual aggression can override the copulatory activity and female receptivity patterns observed in intersexual pairs. When two males are present, the larger male may prevent the smaller, preferred male from interacting with the lone female; when two females are present, the high levels of aggression may preclude the lone male from engaging in copulatory activity. However, these small-group experiments are only the first step in understanding the complex group dynamics in *A. maritima* since this insect species lives in dense aggregations [27,28]. Analyzing earwig social structure through social network analyses would provide a powerful tool in understanding the relative importance of both sex and size on both intrasexual aggression and intersexual mating activity in larger groups. Such analyses could provide powerful insights into how individual characteristics affect large-scale outcomes based on group composition.

## Supporting information

S1 Table Descriptions of behavioral interactions recorded in *A. maritima.* Each behavior is defined by its observable characteristics along with its functional importance.
(DOCX)

S2 Table Results from zero-inflated beta mixed-effect regression models examining the effects of relative size, sex, and their interaction on aggression in (A) Pairs, (B) 2M1F Trios, and (C) 2F1M Trios of *A. maritima* on San Juan Island, WA in 2016. (DOCX)

S3 Table Candidate general linearized models of copulatory activity in pairs of *A. maritima* on San Juan Island in 2016. All models included significant effect of antennation, female total strikes, and a significant interaction between female total strikes and female absolute size.
(DOCX)

S4 Table Candidate general linearized models of copulatory activity in 2F1M Trios of *A. maritima* on San Juan Island in 2016. There were no significant effects of any predictor on the likelihood of copulatory activity occurring.
(DOCX)
