# Supplementary material for: Two’s company, three’s a crowd: Social situations alter group dynamics in the maritime earwig (Anisolabis maritima)
Source: PLoS One. 2026 Mar 10;21(3):e0343830. doi: 10.1371/journal.pone.0343830 (PMC12974818; doi:10.1371/journal.pone.0343830)
Supplement: S1 Table — Each behavior is defined by its observable characteristics along with its functional importance. (DOCX) [file pone.0343830.s001.docx]

**S3 Table. Candidate general linearized models of copulatory activity in pairs of *A. maritima* on San Juan Island in 2016.** All models included significant effect of antennation, female total strikes, and a significant interaction between female total strikes and female absolute size.

| Behavior | Description | Importance |
| --- | --- | --- |
| Antennation | The touching of antennae of two or more individuals | Tactile exchange that serves as a key form of communication in many insects [49] |
| Strike | Rapid and forceful movement characterized by an individual swiftly curving its abdomen in a whip-like motion forward or sideways to bring its forceps into contact with a target where the forceps may also jab or pinch at their target. | Antagonistic interaction that is a key measure of aggression.  Strikes can be either offensive, indicating initiation, or defensive, indicating retaliation. |
| Forceps-to-forceps | Direct contact between the forceps of 2 earwigs facing opposite directions | Part of complex set of behaviors in a mating ritual that is indicative of female receptivity [40, 50] |
| Incidental Contact | Unintentional touching or contact between two or more individuals, often the result of independent movement | Neutral interaction used to gauge overall frequency of interactions |
