# Supplementary material for: Two’s company, three’s a crowd: Social situations alter group dynamics in the maritime earwig (Anisolabis maritima)
Source: PLoS One. 2026 Mar 10;21(3):e0343830. doi: 10.1371/journal.pone.0343830 (PMC12974818; doi:10.1371/journal.pone.0343830)
Supplement: S2 Table — (DOCX) [file pone.0343830.s002.docx]

**S1 Table. Descriptions of behavioral interactions recorded in *A. maritima*.** Each behavior is defined by its observable characteristics along with its functional importance.

| A) Pairs (AIC = 58.032) | | | | | | |
| --- | --- | --- | --- | --- | --- | --- |
| Zero – Inflation Portion | **Coefficient** | **Odds Ratio** | **Estimate** | **SE (Estimate)** | **Z Value** | **P Value** |
|  | Intercept | 0.089 | -2.423 | 0.527 | -4.599 | < 0.001 |
|  | Relative Size | 0.955 | -0.046 | 0.036 | -1.270 | 0.204 |
|  | Sex (Male) | 7.241 | 1.980 | 0.608 | 3.255 | 0.001 |
|  | Rel. Size*Sex | 1.016 | 0.016 | 0.041 | 0.393 | 0.694 |
| Conditional Portion | **Coefficient** | **Odds Ratio** | **Estimate** | **SE (Estimate)** | **Z Value** | **P Value** |
|  | Intercept | 0.3645 | -1.009 | 0.160 | -6.301 | < 0.001 |
|  | Relative Size | 1.028 | 0.028 | 0.008 | 3.492 | < 0.001 |
|  | Sex (Male) | 0.783 | -0.245 | 0.219 | -1.117 | 0.264 |
|  | Rel. Size*Sex | 0.974 | -0.026 | 0.014 | -1.843 | 0.065 |
| B) 2M1F Trios (AIC = 36.747) | | | | | | |
| Zero – Inflation Portion | **Coefficient** | **Odds Ratio** | **Estimate** | **SE (Estimate)** | **Z Value** | **P Value** |
|  | Intercept | 0.371 | -0.992 | 0.295 | -3.357 | < 0.001 |
|  | Relative Size | 1.005 | 0.005 | 0.021 | 0.245 | 0.806 |
|  | Sex (Male) | 2.554 | 0.938 | 0.352 | 2.666 | 0.008 |
|  | Rel. Size*Sex | 0.963 | -0.038 | 0.024 | -1.601 | 0.109 |
| Conditional Portion | **Coefficient** | **Odds Ratio** | **Estimate** | **SE (Estimate)** | **Z Value** | **P Value** |
|  | Intercept | 0.200 | -1.607 | 0.143 | -11.237 | < 0.001 |
|  | Relative Size | 0.999 | -0.001 | 0.009 | -0.113 | 0.910 |
|  | Sex (Male) | 0.629 | -0.464 | 0.152 | -3.058 | 0.002 |
|  | Rel. Size*Sex | 1.013 | 0.013 | 0.011 | 1.195 | 0.232 |
| C) 2F1M Trios (AIC = 80.621) | | | | | | |
| Zero – inflation Portion | **Coefficient** | **Odds Ratio** | **Estimate** | **SE (Estimate)** | **Z Value** | **P Value** |
|  | Intercept | 0.436 | -0.831 | 0.209 | -3.970 | < 0.001 |
|  | Relative Size | 0.947 | -0.054 | 0.014 | -3.939 | < 0.001 |
|  | Sex (Male) | 2.730 | 1.004 | 0.346 | 2.900 | 0.004 |
|  | Rel. Size*Sex | 1.004 | 0.004 | 0.025 | 0.178 | 0.858 |
| Conditional Portion | **Coefficient** | **Odds Ratio** | **Estimate** | **SE (Estimate)** | **Z Value** | **P Value** |
|  | Intercept | 0.250 | -1.386 | 0.160 | -8.686 | < 0.001 |
|  | Relative Size | 1.014 | 0.013 | 0.006 | 2.091 | 0.037 |
|  | Sex (Male) | 0.570 | -0.562 | 0.241 | -2.334 | 0.020 |
|  | Rel. Size*Sex | 1.003 | 0.003 | 0.017 | 0.203 | 0.839 |
