# Supplementary material for: Two’s company, three’s a crowd: Social situations alter group dynamics in the maritime earwig (Anisolabis maritima)
Source: PLoS One. 2026 Mar 10;21(3):e0343830. doi: 10.1371/journal.pone.0343830 (PMC12974818; doi:10.1371/journal.pone.0343830)
Supplement: S3 Table — All models included significant effect of antennation, female total strikes, and a significant interaction between female total strikes and female absolute size. (DOCX) [file pone.0343830.s003.docx]

**S3 Table. Candidate general linearized models of copulatory activity in pairs of *A. maritima* on San Juan Island in 2016.** All models included significant effect of antennation, female total strikes, and a significant interaction between female total strikes and female absolute size.

| A) Pairs – Model 2 (AIC = 53.061) | | | | | |
| --- | --- | --- | --- | --- | --- |
| **Coefficient** | **Odds Ratio** | **Estimate** | **SE (Estimate)** | **Z value** | **P value** |
| Intercept | 0.012 | -4.420 | 1.266 | -3.491 | < 0.001 |
| Antennation | 1.183 | 0.168 | 0.064 | 2.644 | 0.008 |
| Total Strikes (F) | 2.455 | 0.898 | 0.306 | 2.930 | 0.003 |
| Total Strikes (F) * Size (F) | 0.801 | -0.222 | 0.084 | -2.633 | 0.008 |
| B) Pairs – Model 3 (AIC = 53.238) | | | | | |
| **Coefficient** | **Odds Ratio** | **Estimate** | **SE (Estimate)** | **Z value** | **P value** |
| Intercept | < 0.001 | -9.258 | 8.332 | -1.111 | 0.266 |
| Antennation | 1.244 | 0.218 | 0.076 | 2.885 | 0.004 |
| Total Strikes (F) | 3.273 | 1.186 | 0.419 | 2.830 | 0.005 |
| Total Strikes (M) | 1.064 | 0.062 | 0.037 | 1.694 | 0.090 |
| Size (M) | 3.058 | 1.118 | 2.620 | 0.427 | 0.670 |
| Relative Size (F) | 1.099 | 0.095 | 0.078 | 1.215 | 0.224 |
| Total Strikes (F) * Size (F) | 0.734 | -0.310 | 0.121 | -2.556 | 0.011 |
| C) Pairs – Model 4 (AIC = 53.368) | | | | | |
| **Coefficient** | **Odds Ratio** | **Estimate** | **SE (Estimate)** | **Z value** | **P value** |
| Intercept | < 0.001 | -7.759 | 8.522 | -0.910 | 0.363 |
| Antennation | 1.245 | 0.219 | 0.076 | 2.886 | 0.004 |
| Total Strikes (F) | 3.188 | 1.160 | 0.442 | 2.624 | 0.009 |
| Total Strikes (M) | 1.062 | 0.060 | 0.035 | 1.700 | 0.089 |
| Size (F) | 1.878 | 0.630 | 2.700 | 0.233 | 0.815 |
| Relative Size (F) | 1.064 | 0.062 | 0.045 | 1.382 | 0.167 |
| Total Strikes (F) * Size (F) | 0.740 | -0.301 | 0.129 | -2.337 | 0.020 |
| D) Pairs – Model 5 (AIC = 53.704) | | | | | |
| **Coefficient** | **Odds Ratio** | **Estimate** | **SE (Estimate)** | **Z value** | **P value** |
| Intercept | < 0.001 | -8.320 | 8.268 | -1.006 | 0.314 |
| Antennation | 1.240 | 0.215 | 0.075 | 2.875 | 0.004 |
| Total Strikes (F) | 3.293 | 1.192 | 0.443 | 2.692 | 0.007 |
| Total Strikes (M) | 1.059 | 0.058 | 0.034 | 1.672 | 0.094 |
| Size (M) | 0.172 | -1.760 | 1.394 | -1.262 | 0.207 |
| Size (F) | 13.225 | 2.582 | 2.315 | 1.115 | 0.265 |
| Total Strikes (F) * Size (F) | 0.732 | -0.311 | 0.129 | -2.415 | 0.016 |
