# Supplementary material for: Two’s company, three’s a crowd: Social situations alter group dynamics in the maritime earwig (Anisolabis maritima)
Source: PLoS One. 2026 Mar 10;21(3):e0343830. doi: 10.1371/journal.pone.0343830 (PMC12974818; doi:10.1371/journal.pone.0343830)
Supplement: S4 Table — There were no significant effects of any predictor on the likelihood of copulatory activity occurring. (DOCX) [file pone.0343830.s004.docx]

**S4 Table. Candidate general linearized models of copulatory activity in 2F1M Trios of *A. maritima* on San Juan Island in 2016.** There were no significant effects of any predictor on the likelihood of copulatory activity occurring.

| A) 2F1M Trios – Model 2 (AIC = 28.089) | | | | | |
| --- | --- | --- | --- | --- | --- |
| **Coefficient** | **Odds Ratio** | **Estimate** | **SE (Estimate)** | **Z value** | **P value** |
| Intercept | > 100 | 63.550 | 41.328 | 1.538 | 0.124 |
| Size (LF) | > 100 | 92.211 | 88.979 | 1.036 | 0.300 |
| Size (SF) | < 0.001 | -115.284 | 102.178 | -1.128 | 0.259 |
| Relative Size (M to LF) | 16.562 | 2.807 | 2.509 | 1.119 | 0.263 |
| Relative Size (M to SF) | 0.048 | -3.028 | 2.696 | -1.123 | 0.261 |
| Total Strikes (LF to SF) | 0.669 | -0.402 | 0.293 | -1.369 | 0.171 |
| Total Strikes (M to LF) | 1.592 | 0.465 | 0.368 | 1.262 | 0.207 |
| B) 2M1F Trios – Model 3 (AIC = 28.324) | | | | | |
| **Coefficient** | **Odds Ratio** | **Estimate** | **SE (Estimate)** | **Z value** | **P value** |
| Intercept | > 100 | 43.211 | 25.982 | 1.663 | 0.096 |
| Size (LF) | > 100 | 61.953 | 69.302 | 0.894 | 0.371 |
| Size (SF) | < 0.001 | -76.946 | 76.949 | -1.000 | 0.317 |
| Relative size (M to LF) | 7.257 | 1.982 | 1.967 | 1.008 | 0.314 |
| Relative Size (M to SF) | 0.126 | -2.074 | 2.077 | -0.999 | 0.318 |
| Total Strikes (LF to SF) | 0.750 | -0.287 | 0.233 | -1.232 | 0.218 |
| C) 2F1M Trios – Model 4 (AIC = 29.148) | | | | | |
| **Coefficient** | **Odds Ratio** | **Estimate** | **SE (Estimate)** | **Z value** | **P value** |
| Intercept | > 100 | 44.444 | 28.320 | 1.569 | 0.117 |
| Size (LF) | > 100 | 58.764 | 67.102 | 0.876 | 0.381 |
| Size (SF) | < 0.001 | -74.442 | 75.291 | -0.989 | 0.323 |
| Relative Size (M for LF) | 6.998 | 1.946 | 1.893 | 1.028 | 0.304 |
| Relative size (M to SF) | 0.142 | -1.951 | 2.0123 | -0.969 | 0.332 |
| Total Strikes (LF to SF) | 0.588 | -0.531 | 0.312 | -1.701 | 0.089 |
| Total Strikes (M to FL) | 1.336 | 0.290 | 0.317 | 0.916 | 0.360 |
| Relative Size (M to FL) * Total Strikes (LF to SF) | 0.975 | -0.025 | 0.020 | -1.282 | 0.200 |
| D) 2F1M Trios – Model 5 (AIC = 29.277) | | | | | |
| **Coefficient** | **Odds Ratio** | **Estimate** | **SE (Estimate)** | **Z value** | **P value** |
| Intercept | > 100 | 51.208 | 31.609 | 1.620 | 0.105 |
| Size (LF) | > 100 | 99.389 | 85.645 | 1.160 | 0.246 |
| Size (SF) | < 0.001 | -117.495 | 94.997 | -1.237 | 0.216 |
| Relative Size (M to FL) | 4.093 | 1.396 | 2.053 | 0.680 | 0.496 |
| Relative Size (M to SF) | 0.039 | -3.246 | 2.588 | -1.254 | 0.210 |
| Total Strikes (LF to SF) | 0.666 | -0.407 | 0.261 | -1.557 | 0.119 |
| Total Strikes (M to FL) | 1.453 | 0.373 | 0.328 | 1.139 | 0.255 |
| Relative Size (M to FL) * Size (SF) | 1.864 | 0.623 | 0.643 | 0.968 | 0.333 |
